# Investigating the Link between Ketogenic Diet, NAFLD, Mitochondria, and Oxidative Stress: A Narrative Review

**DOI:** 10.3390/antiox12051065

**Published:** 2023-05-08

**Authors:** Antonio Paoli, Giuseppe Cerullo

**Affiliations:** 1 Department of Biomedical Sciences, University of Padova, 35131 Padova, Italy; giuseppe.cerullo@unipd.it; 2 Research Center for High Performance Sport, UCAM Catholic University of Murcia, 30107 Murcia, Spain

**Keywords:** ketogenic diet, nonalcoholic fatty liver disease (NAFLD), mitochondria, oxidative stress, liver, ketone bodies

## Abstract

Together with the global rise in obesity and metabolic syndrome, the prevalence of individuals who suffer from nonalcoholic fatty liver disease (NAFLD) has risen dramatically. NAFLD is currently the most common chronic liver disease and includes a continuum of liver disorders from initial fat accumulation to nonalcoholic steatohepatitis (NASH), considered the more severe forms, which can evolve in, cirrhosis, and hepatocellular carcinoma. Common features of NAFLD includes altered lipid metabolism mainly linked to mitochondrial dysfunction, which, as a vicious cycle, aggravates oxidative stress and promotes inflammation and, as a consequence, the progressive death of hepatocytes and the severe form of NAFLD. A ketogenic diet (KD), i.e., a diet very low in carbohydrates (<30 g/die) that induces “physiological ketosis”, has been demonstrated to alleviate oxidative stress and restore mitochondrial function. Based on this, the aim of the present review is to analyze the body of evidence regarding the potential therapeutic role of KD in NAFLD, focusing on the interplay between mitochondria and the liver, the effects of ketosis on oxidative stress pathways, and the impact of KD on liver and mitochondrial function.

## 1. Introduction

Nonalcoholic fatty liver disease (NAFLD) is the most common chronic liver disease worldwide, with a dramatic increase over the last decade in developing countries [1]. NAFLD covers a wide pathological spectrum of liver injury, from hepatic steatosis (fatty liver) to nonalcoholic steatohepatitis (NASH), recognized as the most serious process, typically characterized by fibrosis, which can culminate in cirrhosis and hepatocellular carcinoma [2]. Essentially, the diagnosis of NAFLD is based on the presence of steatosis in >5% of hepatocytes in the absence of significant ongoing or recent alcohol consumption and other known causes of liver disease [3]. The metabolic impairment associated to this condition, now also called MAFLD (metabolic dysfunction-associated fatty liver disease), may impact both single organelles at cellular level (e.g., mitochondria) and organs and tissue (e.g., liver and adipose tissue) [4]. Noticeably, considering liver’s high metabolic rate, mitochondrial condition is heavily involved in hepatocytes functions. Indeed, liver plays a pivotal role in lipid homeostasis and mitochondria are fundamental in the regulation of hepatic cellular redox, synthesis and catabolism of free fatty acids (FFA) [5]. At the same time, mitochondria produce reactive oxygen species (ROS) which, in case of an imbalance between their overproduction and antioxidant system capacity (oxidative stress: OxS), may lead to a hepatic dysfunction and NAFLD progression [6,7]. Thus, altered lipid metabolism and OxS promote inflammation, mitochondrial dysfunction, fibrosis, and progressive death of hepatocytes [8,9,10]. 

Despite it is unclear if mitochondrial dysfunction should be consider a cause or a consequence of liver disfunction, or both, emerging data shows that hepatic mitochondrial activity may be a potential treatment targets in NAFLD [11]. 

To date, no drugs have been approved for the treatment of NAFLD [12], thus, different nutritional approaches have been explored for the management of NAFLD, but the optimal nutritional therapy remains controversial [13]. At this regard, ketogenic diet (KD), i.e., a diet very low in carbohydrates (<30 g/day) that leads to a “physiological ketosis”, has been demonstrated to improve mitochondrial function and reduce OxS [14,15]. Considering the multiple effects of KD on metabolism [16] it may be used in the treatment of NAFLD, at least at its first stages [17,18,19,20]. 

Considering that ketosis decreases the overall OxS and may slow or reduce mitochondrial dysfunctions, the aim of the present review is to focus on the close link between mitochondria, OxS and liver at the light of the physiology of KD and its potential therapeutic role in NAFLD. 

## 2. NAFLD (in Brief)

In the last decade, there has been a growing interest in investigating NAFLD due to its fast global spread. Today, around one in four adults suffer from this condition and it is estimated that the number of people suffering from NAFLD in the United States will double by 2030 [21]. This rising rate will be followed by increasing numbers of patients with hepatocellular carcinoma, cirrhosis and liver failure. In general, the term NAFLD refers to a wide variety of liver disorders, from simple steatosis, where fat liver infiltration is still relatively low, to nonalcoholic steatohepatitis (NASH), considered the more severe end of the disease spectrum, where liver function (i.e., lipid metabolism) and anatomy (i.e., fibrosis) may be severely compromised [22]. Currently, liver biopsy remains the gold standard method for the definitive diagnosis of NAFL or NASH [23]. In NASH, hepatic steatosis is accompanied by lobular inflammation and exacerbated hepatocyte damage, promoting fibrosis and cirrhosis. Based on level of severity, it’s possible to distinguish from mild fibrosis (stage 1) to bridging fibrosis (stage 4) [1]. 

The NAFLD’s pathogenesis is still not completely understood but the main commonly accepted theories include adipose liver infiltration, abnormalities of hepatocyte metabolism, mitochondria disfunction, altered hepatic immune cell function and systemic inflammation. According to the more simplistic “two hit” hypothesis, the “first hit” derive from insulin resistance and altered lipid metabolism (increased hepatic lipogenesis and impaired FFA degradation), which causes liver steatosis. Thus, this condition sensitizes the liver to further metabolic insults (“second hit”) that led to OxS, activation of inflammation processes and fibrogenesis resulting in the progression of liver disease. More recently, an updated hypothesis has been proposed which involves numerous factors that may act in parallel. This “multiple hits” theory that combined genetic and environmental factors resulting in altered crosstalk between different organs and tissues (e.g., between adipose tissue and other tissues, gut-liver axis, etc.), provides a more appropriate delineation of NAFLD pathogenesis [24,25,26]. 

However, hepatic steatosis is often associated with overweight/obesity (especially the excess of visceral adiposity) or metabolic dysregulation such as type 2 diabetes mellitus, elevated triacylglycerols, decreased high-density lipoprotein cholesterol or increased blood pressure [27,28]. In this scenario, the term MAFLD has recently been suggested to be more appropriate to describe the metabolic dysfunction associated with liver disease [4]. A positive diagnosis of MAFLD can be made in the presence of hepatic steatosis and at least one of the three conditions that follows: overweight or obesity, type 2 diabetes mellitus, and two or more metabolic abnormalities (e.g., high blood pressure, high triglyceridemia, prediabetes, plasma high-sensitivity C-reactive protein level > 2 mg/L) [4].

## 3. Ketogenic Diet: An Overview

The ketogenic diet (KD) is a nutritional pattern characterized by a high content of fat and adequate protein content but a very low carbohydrate intake (less than 20 g d^−1^ or 5% of total daily energy intake) [29]. This macronutrient distribution forces the body to use fat as its primary fuel source, resulting in physiological ketosis (i.e., blood ketone bodies concentrations higher than 0.3 or 0.5 mmol/L and blood pH within the physiological range as a consequence of the increase of ketone bodies (KB) production [30,31]. Growing evidence suggests that carbohydrate-restricted diets, such as KD, can be properly used in several condition from health to disease, i.e., obesity, diabetes, dyslipidemia, hypertension, neurological disorders, and many cancers [30,32,33,34,35]. 

Glucose is widely considered the main energy provider for brain’s metabolism [36]. However, after a period of few days of fasting or KD, glucose storages (glycogen in muscles and liver) become insufficient to support the energy needs of the central nervous system and to sustain fat oxidation process [37]. Indeed, oxaloacetate (an unstable, fundamental intermediate of the Krebs cycle) cannot be accumulated and stored, but must be produced primarily (in mammalians) from the conversion of glucose to pyruvate and, then to oxaloacetate through a, so called, anaplerotic reaction. Despite it is generally accepted that fatty acids cannot cross the blood-brain barrier, some data suggest instead that a certain amount of fatty acids can pass the blood-brain barrier even in a limited quantity [38]. Anyhow, ketone bodies (KBs), i.e., acetoacetate (AcAc), 3-hydroxybutyrate (βHB), and acetone, produced via ketogenesis in the liver mitochondrial matrix from acetyl-CoA, provide an alternate energy source [39]. Depleting glucose, levels of KBs increase to approximately 4 mmol L^−1^ (from <0.3 mmol/L^−1^ under normal circumstances), enhancing the activity of monocarboxylate transporter [40,41]. At this point, KB can be absorbed by the central nervous system as well as numerous different tissues and reconverted to acetyl-CoA, providing a fuel substrate for the Krebs cycle [41]. 

It is critical to emphasize that, despite the deprivation of carbohydrates, blood glucose levels remain physiologically stable due to gluconeogenesis [42], which involves glucogenic amino acids as well (especially in individuals with obesity) as glycerol derived from TGs. In healthy people, this state, where KB levels can rise up to 7 to 8 mmol L^−1^ without any pH change, is considered a physiological adaptation [43]. Instead in the case of pathological diabetic ketoacidosis, extremely high ketonemia (>20 mmol L^−1^), lowering of blood pH (<7.3), and high blood glycemia coexist [44]. 

## 4. KD and Mitochondria

The effect of KBs on mitochondrial function is thought to be among the major contributors to the benefits induced by the KD and the underlying mechanisms are currently under investigation [40,45]. Mitochondria are the most studied organelles in the energy-production system of a cell [46,47], essentially known for ATP generation through the Krebs cycle in the mitochondrial matrix and the oxidative phosphorylation (OXPHOS) in the mitochondrial inner membrane. In addition, mitochondria are even implicated in other vital cell activities such as redox balance, calcium homeostasis, and apoptosis regulation [46,48,49]. It is not unexpected that variations in mitochondrial activity have been associated to a wide range of diseases such as metabolic and degenerative disorders, epilepsy, and cancer [48,50,51,52,53]. Therefore, researchers have increasingly focused on the molecular aspects of mitochondrial dysfunction and strategies for improving mitochondrial activity [11,45,46,51,52]. 

Abnormal mitochondrial functions generally include an impaired OXPHOS, defective mitochondrial dynamics and altered mitochondrial biogenesis strictly linked to a decline in the activity of different regulators of mitochondrial health such as sirtuins (SIRT1-7) or peroxisome proliferator-activated receptor γ (PPARγ) coactivator 1α (PGC-1α) [51]. PGC-1α is commonly considered as a master regulator of biogenesis as well as mitochondrial quality control mechanisms, i.e., mitochondrial remodeling (fission and fusion cycle) and autophagy (mitophagy) [54]; SIRTs are class III nicotinamide-adenine-dinucleotide (NAD+)-dependent histone deacetylases (HDACs) implicated in many signaling that modulate metabolic pathways, redox homeostasis, proliferation, and maintenance of genome stability [55,56]. 

Mitochondria are dynamic structures and their number and function are regulated by the interaction between internal factors and external variables through complex mechanisms. Among external variables, food and physical exercise are the most important factors able to influence mitochondria physiology [57]. 

As underlined by many authors, KD can promote mitochondrial health by improving mitochondrial activity, stimulating the genesis of new mitochondria and remodeling [15,45,57,58,59,60,61]. 

The KD forces the body to use fat as its main source of fuel, as mentioned above. Consequently, the KD inevitably stimulates numerous pathways, upregulating key proteins involved in the OXPHOS system as well as the Krebs cycle (citrate synthase and malate dehydrogenase), FA oxidation (carnitine palmitoyl-transferase, long and very-long chain acyl-coA dehydrogenase and β-hydroxyacyl-coA dehydrogenase) leading to an increase of all these bioenergetic process and mitochondrial activity in general [61]. 

In 2020, Miller and colleagues demonstrated that nutritional ketosis improves mitochondrial efficiency in human skeletal muscle. After 12 weeks on KD combined with exercise, subjects had higher mitochondrial respiratory control ratio (19%, *p* = 0.009) and ATP generation (36%, *p* = 0.028) when compared to the control group (habitual diet and exercise) [59].

Interestingly, KD has been shown to increase the level of mitochondrial uncoupling proteins (UCPs) [62]. Mitochondrial UCPs are a protein family (UCP1–UCP5 in humans) involved in several functions that, due to their pivotal role in regulating the proton gradient across the inner mitochondrial membrane, basically protect mitochondria from oxidative injury and dysfunction [63,64]. In this sense, the impact of KD on mitochondrial bioenergetics could be mediated, at least in part, by the increased activation of mitochondrial UCPs [62]. 

In agreement with these results, Hasan-Olive and colleagues has recently proposed that mitochondrial dysfunction could be reverse via the PGC1α-SIRT3-UCP2 axis activation in mice on a KD [65]. 

During KD or fasting, KB and SIRT1 converge on PGC-1α, acting as direct molecular drivers of epigenomic reprogramming through histone modifications and promoting gluconeogenesis, higher FA oxidation and mitochondrial biogenesis [66,67]. For instance, Wallace and colleagues showed that 14 months of KD increased the expression of the PGC-1α, SIRT1, SIRT3, and proteins from each complex of the electron transport chain, resulting in increases in mitochondrial biogenesis and antioxidant activity concomitant with a mitigation of age-related muscle loss compared to the control group [68]. PGC-1α is implicated in antioxidant response by regulating the expression of many mitochondrial antioxidant genes [69]; indeed, superoxide dismutase-2 (SOD2) and catalase, two antioxidant enzymes, were significantly higher in the KD fed mice, suggesting a positive effect on redox homeostasis [68] (see below).

In general, internal factors that govern mitochondrial biogenesis can be categorized into three levels: (1) those that regulate DNA organelle expression, e.g., mitochondrial transcription factor A (TFAM), which promotes both replication and transcription of the mtDNA; (2) those coordinating nuclear and mitochondrial gene expression, e.g., Nuclear Respiratory Factors (NRF1-2) which control the expression of several nuclear encoded respiratory chain components as well as the expression of TFAM [70]; and (3) those that modulate metabolic process, e.g., PPAR gamma coactivator 1 family of transcription coactivators (PGC-1α) and the SIRTs [70,71]. It seems that KD is able to regulate all 3 levels of regulation of mitochondrial biogenesis, elevating TFAM levels [72,73], activating NRF2 [74], and upregulating SIRTs and PGC-1α. 

Lastly, maintaining mitochondrial morphology is fundamental to preserving or restoring proper function, and KD is associated with better mitochondrial dynamics. Briefly, cycles of fission and fusion are orchestrated mainly by three proteins: MFN1 and MFN2 (mitofusin 1-2) are considered essential for mitochondrial fusion, while DRP1 (dynamin-related protein 1) is required for mitochondrial fission [75,76]. In cases of energy restriction, such as starvation, acute stress, and senescence, increased fusion and/or decreased fission activity promotes mitochondria lengthening [77]. On the other hand, enhanced fission with reduced fusion activity results in the shortening of mitochondria and, thus, impaired bioenergetic activity [77]. Indeed, several conditions caused by excessive food intake, such as type 2 diabetes mellitus or obesity, are characterized by an imbalance in mitochondrial dynamics where smaller and fragmented mitochondria (related to MFN2 downregulation and DRP1 overactivity) are commonly observed [8,78]. Nutrient availability clearly impacts mitochondrial remodeling. Upregulation of MFN2 is recognized as an adaptation to a high-fat diet [77]. As reported by Thai et al., increased βHΒ levels can aid mitochondria repairing process in myocytes from young and aged mice with impaired MFN2-DRP1 axis [79]. KD improves mitochondrial dynamics by preventing mitochondrial fission and inhibiting apoptosis [80]. High levels of βHΒ inhibit mitochondrial fission via suppressing the mitochondrial translocation of DRP1 and, thus, suggesting that KD could have a role in restoring mitochondrial integrity [81].

**Figure 1:**
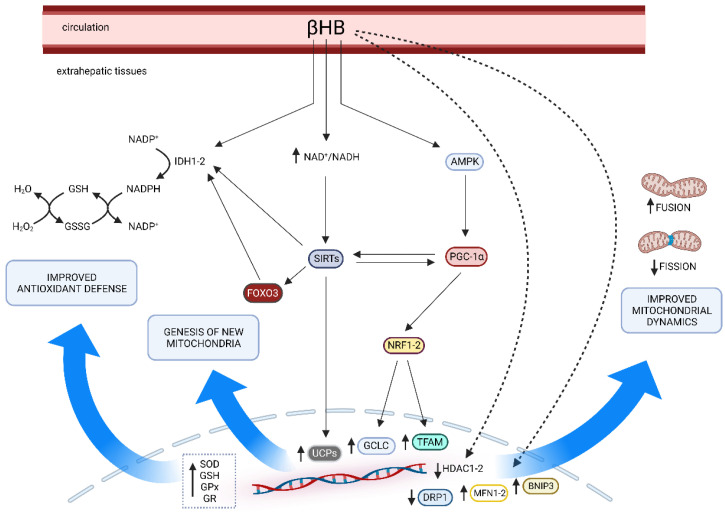
The main pathways involved in the multiple effects of KD on mitochondrial health. The blue arrows suggest the main effects driven by βHB-influenced factors (i.e., improved antioxidant defense, genesis of new mitochondria, and improved mitochondrial dynamics). AMPK, AMP-activated protein kinase; BNIP3, Bcl-2 interacting protein 3; DRP, dynamin related protein 1; GCLC, glutamate cysteine ligase catalytic subunit; GPx, glutathione peroxidase; GR, glutathione reductase; HDAC1-2, histone deacetylases 1-2; IDH1-2, isocitrate dehydrogenase 1-2; MFN2, mitofusin 2; NAD, nicotinamide adenine dinucleotide; NADP, nicotinamide adenine dinucleotide phosphate; NRF1-2, nuclear respiratory factors 1-2; PGC1-α, peroxisome proliferator-activated receptor γ coactivator 1α; SIRTs, sirtuins; SOD, superoxide dismutase; TFAM, mitochondrial transcription factor A; UCPs, mitochondrial uncoupling proteins. Created with BioRender.com, accessed on 25 April 2023.

Furthermore, it seems that KD may even act as a mitophagy activator. Selective elimination of damaged mitochondria via mitochondria-specific autophagy (“mitophagy”) is essential for mitochondrial health, and the expression levels of BNIP3, a mitophagy regulator gene, have been reported to be upregulated in mice fed the KD [82]. Considering the above, it can be suggested that KD may slow or reduce mitochondrial dysfunctions. The multiple effects of KDs (especially βHB) on mitochondrial functions are summarized in Figure 1.

## 5. Effects of Ketosis on Oxidative Stress Pathways

Although it is generally recognized that ketosis decreases the overall OxS, the exact mechanisms are still poorly understood. KB may directly impact OxS; for example, βHB acts as a scavenger for hydroxyl radicals (•OH) due to the presence of the hydroxyl group in βHB [83]. On the other hand, KB could improve the cell’s redox state indirectly in several ways. Among these, studies from Veech and colleagues reported an improvement of mitochondrial function through the increase of the redox span between complex I and II due to decreased reduction of free mitochondrial NAD+, leading to an increased NAD+/NADH ratio and the increase of free mitochondrial CoQ/CoQH ratio [84,85]. During ketosis, an increase in the estimated energy available in the transport of electrons from mitochondrial NAD to Q couple may minimize H_2_O_2_ generation [84]. The positive effects of KB on the OxS, via the increase of NAD+/NADH ratio, has been widely documented in animal [86,87,88,89] ex vivo [90] and cellular models [91,92]. In addition, at constant NADH concentrations, the production of H_2_O_2_ is inhibited by NAD+ [93]. However, there is a close relationship between direct and indirect effects. A paradigmatic example was provided by Milder and colleague demonstrating that in rat model, during a KD, the initial (1 day, 3 days and 1 week) mild increase of OxS (H_2_O_2_) was followed by an activation of NRF2 [74]. NRF2 modulates the expression of various genes involved in the antioxidant response, such as the glutamate cysteine ligase catalytic subunit (GCLC) and modifier subunit (GCLM). GCLC and GCLM compose the heterodimeric protein glutamate cysteine ligase that regulates the production of reduced glutathione (GSH), which is essential for the glutathione peroxidase and thiol-based mitochondrial anti-oxidant system [94]. Other genes and related enzymes are the superoxide dismutase (SOD, EC 1.15.1.1), the catalase (EC 1.11.1.6) and haem oxygenase (HO-1, EC 1.14.99.3), and NAD(P)H:quinone oxidoreductase 1 (Nqo1, EC 1.6.5.2) [95,96].

During ketosis, the enhanced NAD+/NADH ratio is associated with an increased AMP/ATP ratio, due to a rapid ATP depletion, especially under caloric restriction [61]. Thus, the activation of AMPK observed under KD could be related to a high AMP/ATP ratio, at least until compensatory events take place (e.g., improved ATP generation). As proposed by Kolb and colleagues, is ketolysis itself to promote a cellular response, via the activation of AMP-activated protein kinase (AMPK), NRF2, and SIRTs, considered key factors of energetic pathways and other cell-protective activities such as anti-oxidant and anti-inflammatory response, as also stated in the other paragraph [58]. Clearly, KB should be considered not just as a fuel but also as an activator of various signaling pathways that affect energy expenditure, mitochondrial dynamics, and DNA stability [58,97].

Cellular metabolites may also influence gene expression through their activity as cofactors for epigenetic modifications mediated by histones and the effects of KB on OxS could be partially related to those mechanisms associated to the histone acetylation. Inside the cell, transcriptional activity can be modulated by specific epigenome modifiers called histone deacetylases (HDACs, EC 3.5.1.98). Normally, histone acetyltransferase (EC 2.3.1.48) is modulated by nuclear acetyl-CoA concentration whilst NAD+ concentration regulates SIRTs [98]. The higher concentration of Acetil CoA and KB, due to nutritional ketosis of fasting, can influence the acetylation status of histones. Findings from Wang et al. demonstrated a 31–43% inhibition of HDAC activity after 2 weeks of KD in a rat model of spinal cord injury [99]. Similar results have been observed after 10 weeks treatment with βHB in a diabetic rat model [100]. Furthermore, βHB may act as HDAC inhibitor on histone deacetylases of class I and II [101], promoting the expression of FOXO1 and FOXO3a genes and related protein such as HO-1 (FOXO1), SOD2, FOXO3a, and catalase [102]. Nevertheless, βHB has a minimal effect as HDCA inhibitor compared to butyrate [103]. Different results may be attributed to several factors, including the use of in vitro model and different period of fasting/ketosis in the animal model [103,104]. Based on this, it would be speculated that the time under ketosis may have some importance in the HDCA inhibition mediated actions of βHB and a minimum period is needed to obtain the histone-mediated anti-oxidant effects. As previously mentioned, another interesting pathway is the one related to SIRTs. More specifically, SIRT3 exerts many positive effects on OxS by activating antioxidant enzymes such as SOD2 [105], catalase [105], and PGC-1α [106] though the activation of FOXO3a. SIRT3 could also directly modulate SOD2 by deacetylation [107]. 

Finally, the NADPH system also plays an important role in the control of OxS. OxS leads to the accumulation of the oxidized form of glutathione (GSH), glutathione disulfide (GSSG), and, thus, a decreased GSH/GSSG ratio. While in normal fed state NADPH is mainly produced by the hexose monophosphate pathway [108], during KD or fasting, NADPH is produced from KB in the Krebs cycle through the mitochondrial NADP-dependent isocitrate dehydrogenase (IDH2). IDH1 and 2 catalyze the conversion of isocitrate to α-Ketoglutarate in the cytosol and mitochondria, respectively, consuming NADP+ and providing NADPH [109]. NADPH and the combined actions of glutathione peroxidase and glutathione reductase are required for GSH regeneration. Indeed, βHB increases glutathione peroxidase activity (by 4 folds in rat hippocampus) with a positive effect on lipoperoxidation [110] and GSH levels by reducing the NADP/NADPH couple [90,111]. In particular, glutathione reductase is directly dependent on NADPH as an electron donor for the reduction of GSSG [112]. SIRT3 deacetylates IDH2 thus increasing GSH/GSSG ratio [113]. Moreover, both FOXO1 and FOXO3 induce the expression of the IDH1 [114] that regulate the cytoplasmatic production of NADPH from citrate or isocitrate produced in the mitochondria from KBs [115]. 

## 6. Liver and Mitochondria

Mitochondria have a fundamental role in the regulation of hepatic cellular redox metabolism, and its lipid metabolism. In fact, mitochondria occupy about 18% of the hepatocytes volume playing a pivotal role in several signaling pathways linked to fat metabolism [116].

The liver is essential for numerous physiological processes. One of these is lipid metabolism: from digestive absorption (via biliary synthesis and secretion) to FFA homeostasis, including process of synthesis, oxidation, and lipid storage (mainly in form of triglycerides, TG). The liver accumulates FFA in the following ways: (1) by uptaking circulating FFA derived from lipolysis of TG in adipocytes (about 60–80%) and from chylomicron remnant (about 15%); (2) via de novo synthesis of FFA (about 5–25%).

(1) A large part of the FFA pool in the liver derives from the uptake of FFA obtained from adipocytes’ TG through lipolysis. Lipolysis is the process by which TG are hydrolyzed to FFA and glycerol. This process, at the level of adipocytes, involves the sequential action of several lipases, such as adipose TG lipase, hormone-sensitive lipase, and monoglyceride lipase. Then, due to their hydrophobic nature, FFA are transported mainly bounded to albumin from the adipose tissue (storage site) to the other tissues and organs that utilize it (i.e., the liver) [117]. The transport of FFA into the hepatocyte is complex and regulated by specific transporters, including the family of the FFA translocase and the scavenger receptor CD36, and membrane proteins such as caveolins [117].

On the other hand, a small portion of the liver FFA pool is formed from dietary FFA after the digestion process in the small intestine where lipids are emulsified by bile salts. TG are then re-synthesized by enterocytes and transported into the lymph (exocytosis) and blood as lipoprotein particles (chylomicrons with cholesteryl esters, phospholipids, and the apolipoprotein ApoB-48). It was reported that the uptake of chylomicron remnants accounts for up to 25% of the liver FA pool during the fed state [118]. At the hepatocyte, chylomicron remnants are taken up by receptor-mediated endocytosis. Differently, hepatic lipase produces FFA from lipoproteins, which follow the same destiny as plasmatic FFA. In the hepatocyte, FFA could be oxidated or re-esterified (with glycerol) to TG and stored as lipid droplets.

(2) Hepatic de novo lipogenesis is promoted basically by insulin. In fact, both fat and carbohydrates contribute to the FFA pool in the liver. As in other tissues such as in mammary gland, FFA can also be produced from two carbon units (acetyl-CoA). During the process of fatty acid synthesis through acetyl-CoA, mitochondria have a major role: pyruvate, obtained from glucose during glycolysis, can enter the mitochondrion via the mitochondrial pyruvate carrier and, in the matrix, provides Acetyl-CoA, via the pyruvate dehydrogenase complex, and oxaloacetate via the pyruvate carboxylase [119,120]. Nonetheless, pyruvate could be produced from L-lactate through the mitochondrial L-lactate dehydrogenase. Pyruvate and oxaloacetate, involving citrate synthase, may increase citrate in the cytoplasm for FFA synthesis, thereby sustaining the hepatic de novo lipogenesis. Is noteworthy that citrate, the precursor of fatty acid synthesis, is produced in the mitochondrial matrix and subsequently transported outside the mitochondria. In the cytoplasm, where FFA synthesis takes place, the citrate concentration modulates the glycolytic flux by decreasing the activity of phosphofructokinase and promoting the glucose entry in the pentose cycle. This provides NADPH for FFA synthesis. 

Regarding lipid catabolism, firstly, FFA are converted into fatty acyl-CoA by acyl-CoA synthase in the cytosol of hepatocyte. Then, carnitine palmitoyl-transferase 1 catalyzes the reaction from Acyl-CoA + carnitine to CoA and acylcarnitine which can enter mitochondria via the acylcarnitine/L-carnitine antiporter using the L-carnitine shuttle. A second transferase localized at the matrix side of the inner membrane allows the oxidation of acyl-CoA, via the β-oxidation, to acetyl-CoA, while L-carnitine is released. As a result of tricarboxylic acid cycle and oxidative phosphorylation, acetyl-CoA is converted into CO_2_ and water with ATP production [121]. 

Mitochondrial metabolism and hepatocyte energy homeostasis are, therefore, strictly related: indeed, mitochondria are essential in the physiological homeostasis of FFA in hepatocytes as they take part both in the catabolism and in the synthesis processes of the latter [121,122].

The relevance of mitochondrial function in the liver is also being explored in many conditions, from health to illness [122,123,124,125]. Numerous studies have reported altered mitochondrial activity in conditions characterized by liver damage, mainly linked to OxS, poor bioenergetics, and fat accumulation [9,10,124,126]. Overproduction of ROS and lipotoxic lipid accumulation can occur in cases of decreased β-oxidation (e.g., in liver steatosis) or electron transfer chain (ETC) impairments [7]. In more dept, during the early stages of lipid metabolism disturbances, mitochondrial activity boosts, as a compensatory response to minimize the harmful effects of increased lipid accumulation [127]. Nonetheless, the excess of FFA cannot be metabolized completely by hepatic β-oxidation, and consequently, mitochondrial dysfunction that includes changes in oxygen consumption, ETC complex activity and mitochondrial DNA content occurs [7,10,128].

The compromised ETC leads to an overgeneration of ROS, decreased ATP synthesis and the oxidative damage of the internal mitochondrial membrane. This impaired integrity, reduces mitochondrial permeability by opening the mitochondrial permeability transition pore, and allows the release of pro-apoptotic factors to the cytosol (e.g., cytochrome C) [129]. When mitochondria lose cytochrome C, they produce three times as much hydrogen peroxide (H_2_O_2_) than usual [130], causing additional mitochondrial dysfunction and mtDNA mutations promoted by exacerbated OxS [10].

OxS alters ROS signaling pathways, resulting in alterations in mitochondrial biogenesis, mitophagy, and the production of pro-inflammatory (e.g., TNF-α) and pro-fibrosis (e.g., TGF-β) factors [127,131]. Mitochondrial dynamics, biogenesis, and mitophagy determine the mitochondrial population in terms of quality, quantity, and functionality, and are strictly regulated in response to various stressors in order to adjust cellular energetics to metabolic needs [132,133].

Emerging data suggest that mitophagy, a kind of autophagy that targets dysfunctional or superfluous mitochondria, plays a crucial role in the physiology and pathology of the liver [134,135]. Mitophagy preserves mitochondrial bioenergetics and decreases OxS, modulating liver metabolism and protecting it against NAFLD progression [136]. Impaired mitophagy mechanisms cause also an accumulation of highly damaged and aberrant mitochondria, culminating in cell necrosis and the release of bacterial vestiges contained in mitochondria (hypomethylated CpG motifs and formyl-peptides), which may enhance inflammation and development of liver diseases [128]. 

## 7. Liver and Oxidative Stress

ROS generation plays critical roles in normal physiological processes, modulating cellular homeostasis from health to disease [137]. At the same time, an imbalance between the antioxidant system and massive ROS accumulation produces OxS, which promotes an inflammatory response and triggers apoptosis and fibrosis in hepatic tissue [9,138], leading to liver injury and functional dysfunction [26,139]. Generally, mitochondria are recognized as the most important ROS producers and are particularly relevant when considering ROS derived from energetic metabolism [140,141]. Nevertheless, many other sources of non-mitochondrial ROS, have been identified in more recent studies [142]. Among these, peroxisomes that produce H_2_O_2_ as a normal sub-product of fatty acid oxidation [47,143]. Many other enzymes such as NADPH oxidase (NOX), xanthine oxidase, cytochrome P450 (CYP) 2E1, and lipoxygenases even produce ROS, predominantly H_2_O_2_ [144,145]. As recently suggested by several authors, NOX could be considered the major source of ROS production in muscle mass [146,147]. 

OxS is regarded as a crucial factor in the progression of liver disease [9,51,138,148], and mitochondrial dysfunction, endoplasmic reticulum (ER) stress, and NOX up-regulation are basically the principal mechanisms linked to the overproduction of ROS and OxS [6]. Firstly, mitochondrial activity is directly linked to the energy balance and normal function of hepatocytes, as previously mentioned.

At the same time, OxS promotes ER Stress. The ER is an organelle, abundant in hepatocytes, engaged in multiple functions, including lipid metabolism and calcium homeostasis [149]. Moreover, the ER is crucial, especially in the synthesis, folding, and modification of proteins [150]. Alterations in ER redox balance contribute to metabolic dysfunctions and an increase of unfolded protein response (UPR). The UPR is generally controlled by three transmembrane stress transducer proteins: activating transcription factor 6 (ATF6), inositol-requiring signaling protein 1 (IRE1), and protein kinase RNA-like ER kinase (PERK). ER stress causes a variety of consequences in hepatocytes through its downstream pathways. For example, activated IRE1 caused by prolonged UPR activity can prompt apoptosis in two ways: (1) by directly interacting with proapoptotic molecules such as Bax and Bak, and (2) via activating JNK signaling [151]. The ER dysfunction and accumulation of unfolded proteins in the ER can decrease sarco/endoplasmatic reticulum Ca^2+^-ATPase (SERCA) activity, leading to calcium leakage, which blocks the ETC, resulting in the decline of mitochondrial functions and further ROS production [6,10]. The relationship between ER stress, mitochondrial dysfunction, and OxS and its impact on the progression of liver diseases has been deeply investigated by several authors [7,10,152]. In fact, ER stress and OxS are closely linked to the pathogenesis of liver diseases, from simple steatosis to NASH [152].

Finally, the members of NOX family are critical sources of ROS. In the liver, NOX1, NOX2 and NOX4, are expressed in hepatocytes and hepatic stellate cells (HSCs), and NOX2 is expressed by Kupffer cells, which are resident macrophages in the liver [153]. 

Overexpression of NOXs contribute, through the generation of ROS, to oxidative damage and hepatic fibrosis by acting through multiple pathways [154]. For example, the upregulation of NOX-1 mediates NAFLD-induced endothelial dysfunction in the liver. The ROS excess produced by NOX-1 may reduce the NO bioavailability and affect liver circulation by impairing the vasodilation response and decreasing its anti-inflammatory, antifibrogenic, and antioxidant properties in the endothelium [155]. The expression of NOX4 and related ROS generation was significantly increased during development of steatohepatitis in mice [156]. As recently reported by Zhai and colleagues in mouse model of liver injury, NOX4 activates the NLRP3 inflammasome and promotes inflammatory response in KCs by releasing of inflammatory factors, such as IL-6, IL-1β and TNF-α, speculating that NOX4 could be considered as a key factor in inflammatory response [157].

In normal conditions, TNF-α contributes to liver remodeling by driving hepatocyte proliferation and promoting liver regeneration. On the other side, over-production of ROS and consequently OxS is responsible for the prolonged activation of JNK and the further release of TNF-α, which in turn leads to hepatocyte apoptosis [158]. Together, these mechanisms related to OxS can progressively deplete antioxidant capacity and drives various intracellular pathways that contribute to liver dysfunction. Indeed, a reduction of the antioxidant capacity in hepatic cells, such as downregulation of NRF2 and depletion of GSH, is often reported in patients with NAFLD. 

Moreover, OxS contributes to the structural and functional damage of hepatocytes, impairing insulin sensitivity and the activity of key enzymes involved in lipid metabolism [6] and promotes inflammation and the progression of fat accumulation and fibrosis in the liver [6,10]. For example, AMPK signaling is sensitive to OxS. AMPK is a key regulator of cellular metabolism, acting in response to low energy status (↑AMP/ATP) and ROS production, which generally occurs in parallel with bioenergetic processes, can enhance its activity [159]. In order to improve fat oxidation, AMPK reduces the activity of a number of lipid-metabolizing enzymes and nuclear receptors, including acetyl-CoA carboxylase, PPAR, and PPAR, causing a decrease in lipogenesis [160]. Initially, an increase in the AMP/ATP ratio stimulates hepatic AMPK, which limits the NAFL caused by a high-fat diet [161]. However, with progression of NAFLD, despite reductions in liver ATP contents, AMPK is generally repressed, suggesting that chronic exposition to OxS, linked to mitochondrial dysfunction, together with further factors can negatively modulate hepatic AMPK activity [162]. For instance, enhanced inflammatory markers, such as TNF-α, which are common in people with NAFLD, contribute to inhibit the activity of AMPK [163]. Furthermore, AMPK plays an important role in the suppression of proapoptotic caspase-6 protein, and the reduction of AMPK activity relieves this inhibition, promoting hepatocellular death [164].

Although the mechanisms are not fully understood, changes in H_2_O_2_ concentration can affect insulin sensitivity [165,166]. Essentially, both mitochondrial and NOX-derived H_2_O_2_ can promote or suppress insulin sensitivity, and changes in several intracellular signaling pathways have been identified as a major mechanism to explain the relationship between ROS formation and insulin resistance [6].

## 8. KD and Liver

In recent times, the KD has emerged as an effective nutritional strategy for the management of NAFLD and other related metabolic diseases, such as obesity-associated type 2 diabetes mellitus, which plays a pivotal role in the pathogenesis and progression of NAFLD [13,18,19,20]. 

For the first time, a pilot study carried out by Tendler et al. demonstrated that six month of a calorie unrestricted KD led to and improvements in steatosis and fibrosis in four obese patients with histological diagnosis of NAFLD together with a significant weight loss (10.9% on average) [167]. In line with these findings, several authors reported a rapid and marked reduction of liver fat accompanied by a marked decreases in body weight in NAFLD patients treated with KD) [11,168,169,170,171,172]. 

It is crucial to note, however, that in the majority of these studies, there was no control group and KD was combined with caloric restriction. KD and caloric restriction have many pathways and targets in common, as we have underlined in a previous review published in 2019; thus, a synergistic effect can’t be excluded [173].

Considering results from randomized controlled trials, Kirk et al., reported a similar degree of weight loss and intrahepatic TG reduction in obese NAFLD patients comparing results after 11 weeks of KD vs. a control diet equivalent in calories [174]. Similarly, 2-week KD (∼1550 kcal/day) reduced both liver TG (by ~55%) and body weight (−4.6 kg) in obese subjects with NAFLD [175]. In particular, despite similar effects on body weight loss (−4.6 ± 1.5 kg in the KD group vs. −4.0 ± 1.5 kg in the calorie-restricted group), the decrease in hepatic TG content was greater in the KD than in the low-calorie group [175]. 

Overall, based on available evidence, adjusting the dietary macronutrient composition by simply altering the carbohydrate-fat ratio with or without energy intake limitation is recommended [13]. In this regard, KD could be recognized as a promising dietary therapy for NAFLD for several reasons. 

First of all, thanks to their very low carbohydrate content, the KD decrease insulin levels and increase in FA oxidation rate with subsequent reduction in lipogenesis [17,20]. De facto, carbohydrate deprivation is a stimulus for activation of AMPK and SIRT1, even in the absence of caloric restriction. The activation of both SIRT1 and AMPK impacts glucose homeostasis mainly by improving insulin sensitivity [176,177].

As showed by Luukkonen et al., reduction in intrahepatic TG after six days of KD were attributed to increased net hydrolysis of TG and partitioning of the resulting fatty acids toward ketogenesis (+232%) due to reductions in serum insulin concentrations (−53%) and hepatic citrate synthase flux (−38%). Additionally, an increased hepatic mitochondrial redox state (+167%) were observed suggesting hepatic mitochondrial activity as potential treatment targets in NAFLD [11]. As observed in rats, the KD exert anti-steatogenic effects enhancing the liver expression of key genes involved in mitochondrial biogenesis and fatty acid oxidation (PGC-1α and Fgf21) and suppressing inflammatory genes (TNF-α, Nf-kb, and Il-6) [178]. 

Using a multi-omics approach, Mardinoglu and colleagues showed that the improvement of liver fat metabolism in obese adults with NAFLD after a short-term intervention with an isocaloric KD (an average of 3115 kcal/day) was characterized by a rapid decline in numerous inflammatory markers (e.g., IL-6, TNF-α) [179] and even a rapid decrease in plasma concentrations of the peptide hormone fibroblast growth factor 21. Fibroblast growth factor 21 is considered a potential diagnostic marker of NAFLD since its concentrations are higher in NAFLD patients and correlate with hepatic fat content [180,181,182].

**Figure 2:**
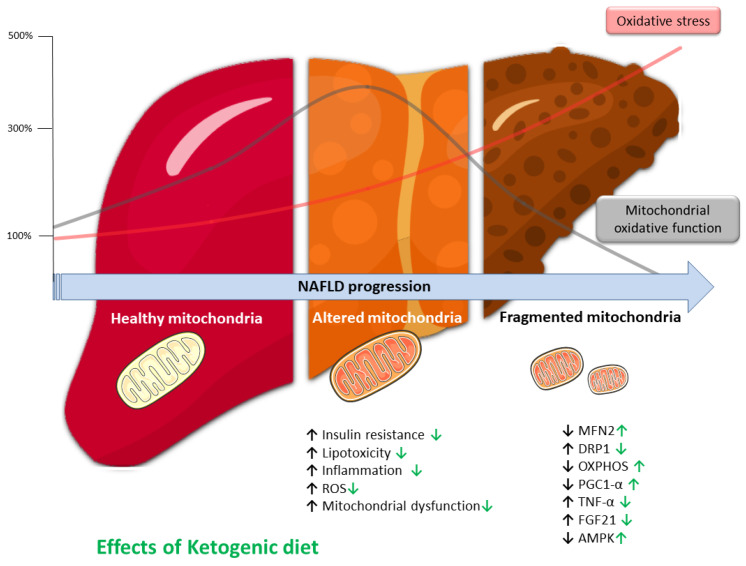
Representation of the link between hepatic mitochondrial adaptation, liver, and oxidative stress during the spectrum of NAFLD. Oxidative capacity can be temporarily enhanced to reduce triglyceride accumulation by increasing fatty acid oxidation. Nonetheless, oxidative stress results in mitochondrial dysfunction and the progression of NAFLD. Chronic oxidative stress stimulates inflammatory pathways, leading to hepatocyte death and liver injury. Up arrows indicate increased and down arrows suggest a decline, while green arrows indicate the effects of a ketogenic diet. AMPK, AMP-activated protein kinase; DRP1, dynamin-related protein 1; FGF21, fibroblast growth factor 21; MFN2, mitofusin 2; OXPHOS, oxidative phosphorylation; PGC1-α, peroxisome proliferator-activated receptor γ coactivator 1α; ROS, reactive oxygen species; TNF-α, tumor necrosis factor-α.

Taken together this body of evidence, severe carbohydrate restriction, as in KD, led favorable outcomes in patients with NAFLD, especially in short and medium term [13]. It is important to underline that the beneficial effects of KD on OxS, inflammation, and mitochondria are mediated by KBs; thus, it seems that it is not carbohydrates restriction per se but rather the complex, multifactorial ketotic metabolic state that might play a role in modulating NAFLD pathophysiology (Figure 2).

Currently, major hepatology associations recommend a weight loss >7–10% in overweight or obese patients with NAFLD because weight reduction is associated with an improvement in histological findings related to liver steatosis, inflammation, and fibrosis [19]. KD resulted in greater long-term reductions in body weight in comparison to a conventional low-fat diet [183]. Nevertheless, at this point, it is unclear if these benefits were caused by weight loss and/or different macronutrient distribution.

Finally, one-fifth of NAFLD patients were classified as lean and 40% were non-obese and, at our knowledge, all published studies have engaged obese or overweight patients [13,19,20]. Further studies enrolling lean subjects with NAFLD are needed to explore potential metabolic benefit in these subjects.

## 9. Conclusions

KD seems to improve mitochondrial dysfunction by stimulating mitochondriogenesis, mitochondrial dynamics, and bioenergetic pathways, as observed especially in vitro and in vivo studies. Ketosis itself plays a pivotal role in stimulating the activation of several key factors involved in liver-protective activities that alleviate oxidative damage, the inflammatory response, and, globally, liver function. However, despite the promising results reported in NAFLD patients, more high-quality, randomized clinical trials are needed.

## Data Availability

Not applicable.

## References

[1] Chalasani N., Younossi Z., Lavine J.E., Charlton M., Cusi K., Rinella M., Harrison S.A., Brunt E.M., Sanyal A.J. (2018). The Diagnosis and Management of Nonalcoholic Fatty Liver Disease: Practice Guidance from the American Association for the Study of Liver Diseases. Hepatology.

[2] Pierantonelli I., Svegliati-Baroni G. (2019). Nonalcoholic Fatty Liver Disease: Basic Pathogenetic Mechanisms in the Progression from NAFLD to NASH. Transplantation.

[3] (2016). EASL–EASD–EASO Clinical Practice Guidelines for the Management of Non-Alcoholic Fatty Liver Disease. J. Hepatol..

[4] Eslam M., Newsome P.N., Sarin S.K., Anstee Q.M., Targher G., Romero-Gomez M., Zelber-Sagi S., Wai-Sun Wong V., Dufour J.-F., Schattenberg J.M. (2020). A New Definition for Metabolic Dysfunction-Associated Fatty Liver Disease: An International Expert Consensus Statement. J. Hepatol..

[5] Li Y., Xie Z., Song Q., Li J. (2022). Mitochondria Homeostasis: Biology and Involvement in Hepatic Steatosis to NASH. Acta Pharm. Sin..

[6] Chen Z., Tian R., She Z., Cai J., Li H. (2020). Role of Oxidative Stress in the Pathogenesis of Nonalcoholic Fatty Liver Disease. Free Radic. Biol. Med..

[7] Gonzalez A., Huerta-Salgado C., Orozco-Aguilar J., Aguirre F., Tacchi F., Simon F., Cabello-Verrugio C. (2020). Role of Oxidative Stress in Hepatic and Extrahepatic Dysfunctions during Nonalcoholic Fatty Liver Disease (NAFLD). Oxid. Med. Cell. Longev..

[8] Longo M., Meroni M., Paolini E., Macchi C., Dongiovanni P. (2021). Mitochondrial Dynamics and Nonalcoholic Fatty Liver Disease (NAFLD): New Perspectives for a Fairy-Tale Ending?. Metabolism.

[9] Cichoz-Lach H., Michalak A. (2014). Oxidative Stress as a Crucial Factor in Liver Diseases. World J. Gastroenterol..

[10] Masarone M., Rosato V., Dallio M., Gravina A.G., Aglitti A., Loguercio C., Federico A., Persico M. (2018). Role of Oxidative Stress in Pathophysiology of Nonalcoholic Fatty Liver Disease. Oxid. Med. Cell. Longev..

[11] Luukkonen P.K., Dufour S., Lyu K., Zhang X.-M., Hakkarainen A., Lehtimäki T.E., Cline G.W., Petersen K.F., Shulman G.I., Yki-Järvinen H. (2020). Effect of a Ketogenic Diet on Hepatic Steatosis and Hepatic Mitochondrial Metabolism in Nonalcoholic Fatty Liver Disease. Proc. Natl. Acad. Sci. USA.

[12] Zhang X.-J., She Z.-G., Li H. (2018). Time to Step-up the Fight against NAFLD. Hepatology.

[13] Parra-Vargas M., Rodriguez-Echevarria R., Jimenez-Chillaron J.C. (2020). Nutritional Approaches for the Management of Nonalcoholic Fatty Liver Disease: An Evidence-Based Review. Nutrients.

[14] Greco T., Glenn T.C., Hovda D.A., Prins M.L. (2016). Ketogenic Diet Decreases Oxidative Stress and Improves Mitochondrial Respiratory Complex Activity. J. Cereb. Blood Flow Metab..

[15] Vidali S., Aminzadeh S., Lambert B., Rutherford T., Sperl W., Kofler B., Feichtinger R.G. (2015). Mitochondria: The Ketogenic Diet—A Metabolism-Based Therapy. Int. J. Biochem. Cell Biol..

[16] Puchalska P., Crawford P.A. (2017). Multi-Dimensional Roles of Ketone Bodies in Fuel Metabolism, Signaling, and Therapeutics. Cell Metab..

[17] Mancin L., Piccini F., Paoli A. (2019). Ketogenic Diet and NAFLD: A Great Therapeutic Opportunity?. Acta Med. Mediterr..

[18] Mooli R.G.R., Ramakrishnan S.K. (2022). Emerging Role of Hepatic Ketogenesis in Fatty Liver Disease. Front. Physiol..

[19] Sripongpun P., Churuangsuk C., Bunchorntavakul C. (2022). Current Evidence Concerning Effects of Ketogenic Diet and Intermittent Fasting in Patients with Nonalcoholic Fatty Liver. J. Clin. Transl. Hepatol..

[20] Watanabe M., Tozzi R., Risi R., Tuccinardi D., Mariani S., Basciani S., Spera G., Lubrano C., Gnessi L. (2020). Beneficial Effects of the Ketogenic Diet on Nonalcoholic Fatty Liver Disease: A Comprehensive Review of the Literature. Obes. Rev..

[21] Estes C., Razavi H., Loomba R., Younossi Z., Sanyal A.J. (2018). Modeling the Epidemic of Nonalcoholic Fatty Liver Disease Demonstrates an Exponential Increase in Burden of Disease. Hepatology.

[22] Sanyal A.J. (2019). Past, Present and Future Perspectives in Nonalcoholic Fatty Liver Disease. Nat. Rev. Gastroenterol. Hepatol..

[23] Siddiqui M.S., Harrison S.A., Abdelmalek M.F., Anstee Q.M., Bedossa P., Castera L., Dimick-Santos L., Friedman S.L., Greene K., Kleiner D.E. (2018). Case Definitions for Inclusion and Analysis of Endpoints in Clinical Trials for Nonalcoholic Steatohepatitis through the Lens of Regulatory Science. Hepatology.

[24] Chen Z., Yu Y., Cai J., Li H. (2019). Emerging Molecular Targets for Treatment of Nonalcoholic Fatty Liver Disease. Trends Endocrinol. Metab..

[25] Friedman S.L., Neuschwander-Tetri B.A., Rinella M., Sanyal A.J. (2018). Mechanisms of NAFLD Development and Therapeutic Strategies. Nat. Med..

[26] Takaki A., Kawai D., Yamamoto K. (2013). Multiple Hits, Including Oxidative Stress, as Pathogenesis and Treatment Target in Non-Alcoholic Steatohepatitis (NASH). Int. J. Mol. Sci..

[27] Bence K.K., Birnbaum M.J. (2021). Metabolic Drivers of Non-Alcoholic Fatty Liver Disease. Mol. Metab..

[28] Ziolkowska S., Binienda A., Jabłkowski M., Szemraj J., Czarny P. (2021). The Interplay between Insulin Resistance, Inflammation, Oxidative Stress, Base Excision Repair and Metabolic Syndrome in Nonalcoholic Fatty Liver Disease. Int. J. Mol. Sci..

[29] Phinney S.D., Bistrian B.R., Evans W.J., Gervino E., Blackburn G.L. (1983). The Human Metabolic Response to Chronic Ketosis without Caloric Restriction: Preservation of Submaximal Exercise Capability with Reduced Carbohydrate Oxidation. Metabolism.

[30] Paoli A., Rubini A., Volek J.S., Grimaldi K.A. (2013). Beyond Weight Loss: A Review of the Therapeutic Uses of Very-Low-Carbohydrate (Ketogenic) Diets. Eur. J. Clin. Nutr..

[31] Ashtary-Larky D., Bagheri R., Bavi H., Baker J.S., Moro T., Mancin L., Paoli A. (2022). Ketogenic Diets, Physical Activity and Body Composition: A Review. Br. J. Nutr..

[32] Paoli A. (2014). Ketogenic Diet for Obesity: Friend or Foe?. Int. J. Environ. Res. Public Health.

[33] Goldenberg J.Z., Johnston B.C. (2021). Low and Very Low Carbohydrate Diets for Diabetes Remission. BMJ.

[34] Harvey C.J.D.C., Schofield G.M., Zinn C., Thornley S.J., Crofts C., Merien F.L.R. (2019). Low-Carbohydrate Diets Differing in Carbohydrate Restriction Improve Cardiometabolic and Anthropometric Markers in Healthy Adults: A Randomised Clinical Trial. PeerJ.

[35] Ilyas Z., Perna S., Alalwan T.A., Zahid M.N., Spadaccini D., Gasparri C., Peroni G., Faragli A., Alogna A., la Porta E. (2022). The Ketogenic Diet: Is It an Answer for Sarcopenic Obesity?. Nutrients.

[36] Robinson P.J., Rapoport S.I. (1986). Glucose Transport and Metabolism in the Brain. Am. J. Physiol. Regul. Integr. Comp. Physiol..

[37] Owen O.E., Morgan A.P., Kemp H.G., Sullivan J.M., Herrera M.G., Cahill G.F. (1967). Brain Metabolism during Fasting. J. Clin. Investig..

[38] Mitchell R.W., Hatch G.M. (2011). Fatty Acid Transport into the Brain: Of Fatty Acid Fables and Lipid Tails. Prostaglandins Leukot. Essent. Fat. Acids.

[39] McGarry J.D., Foster D.W. (1976). Ketogenesis and Its Regulation. Am. J. Med..

[40] Veech R.L. (2004). The Therapeutic Implications of Ketone Bodies: The Effects of Ketone Bodies in Pathological Conditions: Ketosis, Ketogenic Diet, Redox States, Insulin Resistance, and Mitochondrial Metabolism. Prostaglandins Leukot. Essent. Fat. Acids.

[41] Leino R.L., Gerhart D.Z., Duelli R., Enerson B.E., Drewes L.R. (2001). Diet-Induced Ketosis Increases Monocarboxylate Transporter (MCT1) Levels in Rat Brain. Neurochem. Int..

[42] Paoli A., Cenci L., Grimaldi K.A. (2011). Effect of Ketogenic Mediterranean Diet with Phytoextracts and Low Carbohydrates/High-Protein Meals on Weight, Cardiovascular Risk Factors, Body Composition and Diet Compliance in Italian Council Employees. Nutr. J..

[43] Krebs H.A. (1966). The Regulation of the Release of Ketone Bodies by the Liver. Adv. Enzym. Regul..

[44] Paoli A., Bianco A., Grimaldi K.A. (2015). The Ketogenic Diet and Sport. Exerc. Sport Sci. Rev..

[45] Pathak S.J., Baar K. (2023). Ketogenic Diets and Mitochondrial Function: Benefits for Aging But Not for Athletes. Exerc. Sport Sci. Rev..

[46] Akbari M., Kirkwood T.B.L., Bohr V.A. (2019). Mitochondria in the Signaling Pathways That Control Longevity and Health Span. Ageing Res. Rev..

[47] Negro M., Cerullo G., Parimbelli M., Ravazzani A., Feletti F., Berardinelli A., Cena H., D’Antona G. (2021). Exercise, Nutrition, and Supplements in the Muscle Carnitine Palmitoyl-Transferase II Deficiency: New Theoretical Bases for Potential Applications. Front. Physiol..

[48] Vásquez-Trincado C., García-Carvajal I., Pennanen C., Parra V., Hill J.A., Rothermel B.A., Lavandero S. (2016). Mitochondrial Dynamics, Mitophagy and Cardiovascular Disease. J. Physiol..

[49] Nunnari J., Suomalainen A. (2012). Mitochondria: In Sickness and in Health. Cell.

[50] Forbes J.M., Thorburn D.R. (2018). Mitochondrial Dysfunction in Diabetic Kidney Disease. Nat. Rev. Nephrol..

[51] Sorrentino V., Menzies K.J., Auwerx J. (2018). Repairing Mitochondrial Dysfunction in Disease. Annu. Rev. Pharm. Toxicol..

[52] Rai S.N., Singh C., Singh A., Singh M.P., Singh B.K. (2020). Mitochondrial Dysfunction: A Potential Therapeutic Target to Treat Alzheimer’s Disease. Mol. Neurobiol..

[53] Chistiakov D.A., Shkurat T.P., Melnichenko A.A., Grechko A.v., Orekhov A.N. (2018). The Role of Mitochondrial Dysfunction in Cardiovascular Disease: A Brief Review. Ann. Med..

[54] Halling J.F., Pilegaard H. (2020). PGC-1α-Mediated Regulation of Mitochondrial Function and Physiological Implications. Appl. Physiol. Nutr. Metab..

[55] Singh C.K., Chhabra G., Ndiaye M.A., Garcia-Peterson L.M., Mack N.J., Ahmad N. (2018). The Role of Sirtuins in Antioxidant and Redox Signaling. Antioxid. Redox Signal..

[56] Imai S., Guarente L. (2014). NAD+ and Sirtuins in Aging and Disease. Trends Cell Biol..

[57] Kyriazis I., Vassi E., Alvanou M., Angelakis C., Skaperda Z., Tekos F., Garikipati V., Spandidos D., Kouretas D. (2022). The Impact of Diet upon Mitochondrial Physiology (Review). Int. J. Mol. Med..

[58] Kolb H., Kempf K., Röhling M., Lenzen-Schulte M., Schloot N.C., Martin S. (2021). Ketone Bodies: From Enemy to Friend and Guardian Angel. BMC Med..

[59] Miller V.J., LaFountain R.A., Barnhart E., Sapper T.S., Short J., Arnold W.D., Hyde P.N., Crabtree C.D., Kackley M.L., Kraemer W.J. (2020). A Ketogenic Diet Combined with Exercise Alters Mitochondrial Function in Human Skeletal Muscle While Improving Metabolic Health. Am. J. Physiol. Endocrinol. Metab..

[60] Qu C., Keijer J., Adjobo-Hermans M.J.W., van de Wal M., Schirris T., van Karnebeek C., Pan Y., Koopman W.J.H. (2021). The Ketogenic Diet as a Therapeutic Intervention Strategy in Mitochondrial Disease. Int. J. Biochem. Cell Biol..

[61] Miller V.J., Villamena F.A., Volek J.S. (2018). Nutritional Ketosis and Mitohormesis: Potential Implications for Mitochondrial Function and Human Health. J. Nutr. Metab..

[62] Sullivan P.G., Rippy N.A., Dorenbos K., Concepcion R.C., Agarwal A.K., Rho J.M. (2004). The Ketogenic Diet Increases Mitochondrial Uncoupling Protein Levels and Activity. Ann. Neurol..

[63] Wolkow C.A., Iser W.B. (2006). Uncoupling Protein Homologs May Provide a Link between Mitochondria, Metabolism and Lifespan. Ageing Res. Rev..

[64] Demine S., Renard P., Arnould T. (2019). Mitochondrial Uncoupling: A Key Controller of Biological Processes in Physiology and Diseases. Cells.

[65] Hasan-Olive M.M., Lauritzen K.H., Ali M., Rasmussen L.J., Storm-Mathisen J., Bergersen L.H. (2019). A Ketogenic Diet Improves Mitochondrial Biogenesis and Bioenergetics via the PGC1α-SIRT3-UCP2 Axis. Neurochem. Res..

[66] Abduraman M.A., Azizan N.A., Teoh S.H., Tan M.L. (2021). Ketogenesis and SIRT1 as a Tool in Managing Obesity. Obes. Res. Clin. Pract..

[67] Tozzi R., Cipriani F., Masi D., Basciani S., Watanabe M., Lubrano C., Gnessi L., Mariani S. (2022). Ketone Bodies and SIRT1, Synergic Epigenetic Regulators for Metabolic Health: A Narrative Review. Nutrients.

[68] Wallace M.A., Aguirre N.W., Marcotte G.R., Marshall A.G., Baehr L.M., Hughes D.C., Hamilton K.L., Roberts M.N., Lopez-Dominguez J.A., Miller B.F. (2021). The Ketogenic Diet Preserves Skeletal Muscle with Aging in Mice. Aging Cell.

[69] Rius-Pérez S., Torres-Cuevas I., Millán I., Ortega Á.L., Pérez S., Sandhu M.A. (2020). PGC-1 α, Inflammation, and Oxidative Stress: An Integrative View in Metabolism. Oxid. Med. Cell. Longev..

[70] Carelli V., Maresca A., Caporali L., Trifunov S., Zanna C., Rugolo M. (2015). Mitochondria: Biogenesis and Mitophagy Balance in Segregation and Clonal Expansion of Mitochondrial DNA Mutations. Int. J. Biochem. Cell Biol..

[71] Popov L. (2020). Mitochondrial Biogenesis: An Update. J. Cell. Mol. Med..

[72] Jornayvaz F.R., Jurczak M.J., Lee H.-Y., Birkenfeld A.L., Frederick D.W., Zhang D., Zhang X.-M., Samuel V.T., Shulman G.I. (2010). A High-Fat, Ketogenic Diet Causes Hepatic Insulin Resistance in Mice, despite Increasing Energy Expenditure and Preventing Weight Gain. Am. J. Physiol. Endocrinol. Metab..

[73] Moore M.P., Cunningham R.P., Kelty T.J., Boccardi L.R., Nguyen N.Y., Booth F.W., Rector R.S. (2020). Ketogenic Diet in Combination with Voluntary Exercise Impacts Markers of Hepatic Metabolism and Oxidative Stress in Male and Female Wistar Rats. Appl. Physiol. Nutr. Metab..

[74] Milder J.B., Liang L.-P., Patel M. (2010). Acute Oxidative Stress and Systemic Nrf2 Activation by the Ketogenic Diet. Neurobiol. Dis..

[75] Tilokani L., Nagashima S., Paupe V., Prudent J. (2018). Mitochondrial Dynamics: Overview of Molecular Mechanisms. Essays Biochem..

[76] Sidarala V., Zhu J., Levi-D’Ancona E., Pearson G.L., Reck E.C., Walker E.M., Kaufman B.A., Soleimanpour S.A. (2022). Mitofusin 1 and 2 Regulation of Mitochondrial DNA Content Is a Critical Determinant of Glucose Homeostasis. Nat. Commun..

[77] Liesa M., Shirihai O.S. (2013). Mitochondrial Dynamics in the Regulation of Nutrient Utilization and Energy Expenditure. Cell Metab..

[78] Williams M., Caino M.C. (2018). Mitochondrial Dynamics in Type 2 Diabetes and Cancer. Front. Endocrinol..

[79] Thai P.N., Seidlmayer L.K., Miller C., Ferrero M., Dorn G.W., Schaefer S., Bers D.M., Dedkova E.N. (2019). Mitochondrial Quality Control in Aging and Heart Failure: Influence of Ketone Bodies and Mitofusin-Stabilizing Peptides. Front. Physiol..

[80] Guo Y., Zhang C., Shang F.-F., Luo M., You Y., Zhai Q., Xia Y., Suxin L. (2020). Ketogenic Diet Ameliorates Cardiac Dysfunction via Balancing Mitochondrial Dynamics and Inhibiting Apoptosis in Type 2 Diabetic Mice. Aging Dis..

[81] Guo M., Wang X., Zhao Y., Yang Q., Ding H., Dong Q., Chen X., Cui M. (2018). Ketogenic Diet Improves Brain Ischemic Tolerance and Inhibits NLRP3 Inflammasome Activation by Preventing Drp1-Mediated Mitochondrial Fission and Endoplasmic Reticulum Stress. Front. Mol. Neurosci..

[82] Newell C., Shutt T.E., Ahn Y., Hittel D.S., Khan A., Rho J.M., Shearer J. (2016). Tissue Specific Impacts of a Ketogenic Diet on Mitochondrial Dynamics in the BTBRT+tf/j Mouse. Front. Physiol..

[83] Haces M.L., Hernández-Fonseca K., Medina-Campos O.N., Montiel T., Pedraza-Chaverri J., Massieu L. (2008). Antioxidant Capacity Contributes to Protection of Ketone Bodies against Oxidative Damage Induced during Hypoglycemic Conditions. Exp. Neurol..

[84] Sato K., Kashiwaya Y., Keon C.A., Tsuchiya N., King M.T., Radda G.K., Chance B., Clarke K., Veech R.L. (1995). Insulin, Ketone Bodies, and Mitochondrial Energy Transduction. FASEB J..

[85] Veech R.L. (2006). The Determination of the Redox States and Phosphorylation Potential in Living Tissues and Their Relationship to Metabolic Control of Disease Phenotypes. Biochem. Mol. Biol. Educ..

[86] Pawlosky R.J., Kemper M.F., Kashiwaya Y., King M.T., Mattson M.P., Veech R.L. (2017). Effects of a Dietary Ketone Ester on Hippocampal Glycolytic and Tricarboxylic Acid Cycle Intermediates and Amino Acids in a 3xTgAD Mouse Model of Alzheimer’s Disease. J. Neurochem..

[87] Xin L., Ipek Ö., Beaumont M., Shevlyakova M., Christinat N., Masoodi M., Greenberg N., Gruetter R., Cuenoud B. (2018). Nutritional Ketosis Increases NAD+/NADH Ratio in Healthy Human Brain: An in Vivo Study by 31P-MRS. Front. Nutr..

[88] Yin J., Han P., Tang Z., Liu Q., Shi J. (2015). Sirtuin 3 Mediates Neuroprotection of Ketones against Ischemic Stroke. J. Cereb. Blood Flow Metab..

[89] Yin J., Nielsen M., Li S., Shi J. (2019). Ketones Improves Apolipoprotein E4-Related Memory Deficiency via Sirtuin 3. Aging.

[90] Kashiwaya Y., King M.T., Veech R.L. (1997). Substrate Signaling by Insulin. Am. J. Cardiol..

[91] Maalouf M., Sullivan P.G., Davis L., Kim D.Y., Rho J.M. (2007). Ketones Inhibit Mitochondrial Production of Reactive Oxygen Species Production Following Glutamate Excitotoxicity by Increasing NADH Oxidation. Neuroscience.

[92] Marosi K., Kim S.W., Moehl K., Scheibye-Knudsen M., Cheng A., Cutler R., Camandola S., Mattson M.P. (2016). 3-Hydroxybutyrate Regulates Energy Metabolism and Induces BDNF Expression in Cerebral Cortical Neurons. J. Neurochem..

[93] Hirst J., King M.S., Pryde K.R. (2008). The Production of Reactive Oxygen Species by Complex I. Biochem. Soc. Trans..

[94] Franklin C.C., Backos D.S., Mohar I., White C.C., Forman H.J., Kavanagh T.J. (2009). Structure, Function, and Post-Translational Regulation of the Catalytic and Modifier Subunits of Glutamate Cysteine Ligase. Mol. Asp. Med..

[95] Hayes J.D., Dinkova-Kostova A.T. (2014). The Nrf2 Regulatory Network Provides an Interface between Redox and Intermediary Metabolism. Trends Biochem. Sci..

[96] Tebay L.E., Robertson H., Durant S.T., Vitale S.R., Penning T.M., Dinkova-Kostova A.T., Hayes J.D. (2015). Mechanisms of Activation of the Transcription Factor Nrf2 by Redox Stressors, Nutrient Cues, and Energy Status and the Pathways through Which It Attenuates Degenerative Disease. Free Radic. Biol. Med..

[97] Miller A.L. (1999). Therapeutic Considerations of L-Glutamine: A Review of the Literature. Altern. Med. Rev..

[98] Pasyukova E.G., Vaiserman A.M. (2017). HDAC Inhibitors: A New Promising Drug Class in Anti-Aging Research. Mech. Ageing Dev..

[99] Wang X., Wu X., Liu Q., Kong G., Zhou J., Jiang J., Wu X., Huang Z., Su W., Zhu Q. (2017). Ketogenic Metabolism Inhibits Histone Deacetylase (HDAC) and Reduces Oxidative Stress After Spinal Cord Injury in Rats. Neuroscience.

[100] Li B., Yu Y., Liu K., Zhang Y., Geng Q., Zhang F., Li Y., Qi J. (2021). β-Hydroxybutyrate Inhibits Histone Deacetylase 3 to Promote Claudin-5 Generation and Attenuate Cardiac Microvascular Hyperpermeability in Diabetes. Diabetologia.

[101] Shimazu T., Hirschey M.D., Newman J., He W., Shirakawa K., le Moan N., Grueter C.A., Lim H., Saunders L.R., Stevens R.D. (2013). Suppression of Oxidative Stress by β-Hydroxybutyrate, an Endogenous Histone Deacetylase Inhibitor. Science.

[102] Rojas-Morales P., Pedraza-Chaverri J., Tapia E. (2020). Ketone Bodies, Stress Response, and Redox Homeostasis. Redox Biol..

[103] Chriett S., Dąbek A., Wojtala M., Vidal H., Balcerczyk A., Pirola L. (2019). Prominent Action of Butyrate over β-Hydroxybutyrate as Histone Deacetylase Inhibitor, Transcriptional Modulator and Anti-Inflammatory Molecule. Sci. Rep..

[104] Xie Z., Zhang D., Chung D., Tang Z., Huang H., Dai L., Qi S., Li J., Colak G., Chen Y. (2016). Metabolic Regulation of Gene Expression by Histone Lysine β-Hydroxybutyrylation. Mol. Cell.

[105] Qiu X., Brown K., Hirschey M.D., Verdin E., Chen D. (2010). Calorie Restriction Reduces Oxidative Stress by SIRT3-Mediated SOD2 Activation. Cell Metab..

[106] Weng H., Ma Y., Chen L., Cai G., Chen Z., Zhang S., Ye Q. (2020). A New Vision of Mitochondrial Unfolded Protein Response to the Sirtuin Family. Curr. Neuropharmacol..

[107] Chen Y., Zhang J., Lin Y., Lei Q., Guan K., Zhao S., Xiong Y. (2011). Tumour Suppressor SIRT3 Deacetylates and Activates Manganese Superoxide Dismutase to Scavenge ROS. EMBO Rep..

[108] Stincone A., Prigione A., Cramer T., Wamelink M.M.C., Campbell K., Cheung E., Olin-Sandoval V., Grüning N., Krüger A., Tauqeer Alam M. (2015). The Return of Metabolism: Biochemistry and Physiology of the Pentose Phosphate Pathway. Biol. Rev..

[109] White K., Someya S. (2023). The Roles of NADPH and Isocitrate Dehydrogenase in Cochlear Mitochondrial Antioxidant Defense and Aging. Hear. Res..

[110] Ziegler D.R., Ribeiro L.C., Hagenn M., Siqueira I.R., Araújo E., Torres I.L.S., Gottfried C., Netto C.A., Gonçalves C. (2003). Ketogenic Diet Increases Glutathione Peroxidase Activity in Rat Hippocampus. Neurochem. Res..

[111] Vogel R., Wiesinger H., Hamprecht B., Dringen R. (1999). The Regeneration of Reduced Glutathione in Rat Forebrain Mitochondria Identifies Metabolic Pathways Providing the NADPH Required. Neurosci. Lett..

[112] Garcia J., Han D., Sancheti H., Yap L.-P., Kaplowitz N., Cadenas E. (2010). Regulation of Mitochondrial Glutathione Redox Status and Protein Glutathionylation by Respiratory Substrates. J. Biol. Chem..

[113] Noh M.R., Kong M.J., Han S.J., Kim J.I., Park K.M. (2020). Isocitrate Dehydrogenase 2 Deficiency Aggravates Prolonged High-Fat Diet Intake-Induced Hypertension. Redox Biol..

[114] Charitou P., Rodriguez-Colman M., Gerrits J., Triest M., Groot Koerkamp M., Hornsveld M., Holstege F., Verhoeven-Duif N.M., Burgering B.M. (2015). FOXOs Support the Metabolic Requirements of Normal and Tumor Cells by Promoting IDH1 Expression. EMBO Rep..

[115] Veech R.L., Bradshaw P.C., Clarke K., Curtis W., Pawlosky R., King M.T. (2017). Ketone Bodies Mimic the Life Span Extending Properties of Caloric Restriction. IUBMB Life.

[116] Guerrieri F., Nicoletti C., Adorisio E., Caraccio G., Leonetti P., Zanotti F., Cantatore P. (2000). Correlation between Decreased Expression of Mitochondrial F0F1-ATP Synthase and Low Regenerating Capability of the Liver after Partial Hepatectomy in Hypothyroid Rats. J. Bioenerg. Biomembr..

[117] Mashek D.G. (2013). Hepatic Fatty Acid Trafficking: Multiple Forks in the Road. Adv. Nutr..

[118] Barrows B.R., Parks E.J. (2006). Contributions of Different Fatty Acid Sources to Very Low-Density Lipoprotein-Triacylglycerol in the Fasted and Fed States. J. Clin. Endocrinol. Metab..

[119] Passarella S., de Bari L., Valenti D., Pizzuto R., Paventi G., Atlante A. (2008). Mitochondria and L-Lactate Metabolism. FEBS Lett..

[120] Paventi G., Pizzuto R., Passarella S. (2017). The Occurrence of L-Lactate Dehydrogenase in the Inner Mitochondrial Compartment of Pig Liver. Biochem. Biophys. Res. Commun..

[121] Tamura Y., Kawano S., Endo T. (2020). Lipid Homeostasis in Mitochondria. Biol. Chem..

[122] Fromenty B., Roden M. (2023). Mitochondrial Alterations in Fatty Liver Diseases. J. Hepatol..

[123] Zhang C., Zhao Y., Yu M., Qin J., Ye B., Wang Q. (2022). Mitochondrial Dysfunction and Chronic Liver Disease. Curr. Issues Mol. Biol..

[124] Di Ciaula A., Passarella S., Shanmugam H., Noviello M., Bonfrate L., Wang D.Q.-H., Portincasa P. (2021). Nonalcoholic Fatty Liver Disease (NAFLD). Mitochondria as Players and Targets of Therapies?. Int. J. Mol. Sci..

[125] Grattagliano I., de Bari O., Bernardo T.C., Oliveira P.J., Wang D.Q.-H., Portincasa P. (2012). Role of Mitochondria in Nonalcoholic Fatty Liver Disease-from Origin to Propagation. Clin. Biochem..

[126] Muriel P., Gordillo K.R. (2016). Role of Oxidative Stress in Liver Health and Disease. Oxid. Med. Cell. Longev..

[127] Ott M., Gogvadze V., Orrenius S., Zhivotovsky B. (2007). Mitochondria, Oxidative Stress and Cell Death. Apoptosis.

[128] Mansouri A., Gattolliat C.H., Asselah T. (2018). Mitochondrial Dysfunction and Signaling in Chronic Liver Diseases. Gastroenterology.

[129] Simões I.C.M., Fontes A., Pinton P., Zischka H., Wieckowski M.R. (2018). Mitochondria in Non-Alcoholic Fatty Liver Disease. Int. J. Biochem. Cell Biol..

[130] Kushnareva Y., Murphy A.N., Andreyev A. (2002). Complex I-Mediated Reactive Oxygen Species Generation: Modulation by Cytochrome c and NAD(P)+ Oxidation-Reduction State. Biochem. J..

[131] Fischer R., Maier O. (2015). Interrelation of Oxidative Stress and Inflammation in Neurodegenerative Disease: Role of TNF. Oxid. Med. Cell. Longev..

[132] Romanello V., Sandri M. (2021). The Connection between the Dynamic Remodeling of the Mitochondrial Network and the Regulation of Muscle Mass. Cell. Mol. Life Sci..

[133] Romanello V., Sandri M. (2023). Implications of Mitochondrial Fusion and Fission in Skeletal Muscle Mass and Health. Semin. Cell Dev. Biol..

[134] Ma X., McKeen T., Zhang J., Ding W.-X. (2020). Role and Mechanisms of Mitophagy in Liver Diseases. Cells.

[135] Ramanathan R., Ali A.H., Ibdah J.A. (2022). Mitochondrial Dysfunction Plays Central Role in Nonalcoholic Fatty Liver Disease. Int. J. Mol. Sci..

[136] Madrigal-Matute J., Cuervo A.M. (2016). Regulation of Liver Metabolism by Autophagy. Gastroenterology.

[137] Zuo J., Zhang Z., Luo M., Zhou L., Nice E.C., Zhang W., Wang C., Huang C. (2022). Redox Signaling at the Crossroads of Human Health and Disease. MedComm.

[138] Li S., Tan H.Y., Wang N., Zhang Z.J., Lao L., Wong C.W., Feng Y. (2015). The Role of Oxidative Stress and Antioxidants in Liver Diseases. Int. J. Mol. Sci..

[139] Rolo A.P., Teodoro J.S., Palmeira C.M. (2012). Role of Oxidative Stress in the Pathogenesis of Nonalcoholic Steatohepatitis. Free Radic. Biol. Med..

[140] Figueira T.R., Barros M.H., Camargo A.A., Castilho R.F., Ferreira J.C.B., Kowaltowski A.J., Sluse F.E., Souza-Pinto N.C., Vercesi A.E. (2013). Mitochondria as a Source of Reactive Oxygen and Nitrogen Species: From Molecular Mechanisms to Human Health. Antioxid. Redox Signal..

[141] Starkov A.A. (2008). The Role of Mitochondria in Reactive Oxygen Species Metabolism and Signaling. Ann. N. Y. Acad. Sci..

[142] Zhang Y., Wong H.S. (2021). Are Mitochondria the Main Contributor of Reactive Oxygen Species in Cells?. J. Exp. Biol..

[143] del Río L.A., López-Huertas E. (2016). ROS Generation in Peroxisomes and Its Role in Cell Signaling. Plant Cell Physiol..

[144] Forrester S.J., Kikuchi D.S., Hernandes M.S., Xu Q., Griendling K.K. (2018). Reactive Oxygen Species in Metabolic and Inflammatory Signaling. Circ. Res..

[145] Henríquez-Olguín C., Boronat S., Cabello-Verrugio C., Jaimovich E., Hidalgo E., Jensen T.E. (2019). The Emerging Roles of Nicotinamide Adenine Dinucleotide Phosphate Oxidase 2 in Skeletal Muscle Redox Signaling and Metabolism. Antioxid. Redox Signal..

[146] Powers S.K., Deminice R., Ozdemir M., Yoshihara T., Bomkamp M.P., Hyatt H. (2020). Exercise-Induced Oxidative Stress: Friend or Foe?. J. Sport Health Sci..

[147] Henriquez-Olguin C., Meneses-Valdes R., Jensen T.E. (2020). Compartmentalized Muscle Redox Signals Controlling Exercise Metabolism–Current State, Future Challenges. Redox Biol..

[148] Schieber M., Chandel N.S. (2014). ROS Function in Redox Signaling and Oxidative Stress. Curr. Biol..

[149] Zheng W., Sun Q., Li L., Cheng Y., Chen Y., Lv M., Xiang X. (2022). Role of Endoplasmic Reticulum Stress in Hepatic Glucose and Lipid Metabolism and Therapeutic Strategies for Metabolic Liver Disease. Int. Immunopharmacol..

[150] Csordás G., Weaver D., Hajnóczky G. (2018). Endoplasmic Reticulum-Mitochondrial Contactology: Structure and Signaling Functions. Trends Cell Biol..

[151] Lebeaupin C., Vallée D., Hazari Y., Hetz C., Chevet E., Bailly-Maitre B. (2018). Endoplasmic Reticulum Stress Signalling and the Pathogenesis of Non-Alcoholic Fatty Liver Disease. J. Hepatol..

[152] Ashraf N.U., Sheikh T.A. (2015). Endoplasmic Reticulum Stress and Oxidative Stress in the Pathogenesis of Non-Alcoholic Fatty Liver Disease. Free Radic. Res..

[153] Herranz-Itúrbide M., Peñuelas-Haro I., Espinosa-Sotelo R., Bertran E., Fabregat I. (2021). The TGF-β/NADPH Oxidases Axis in the Regulation of Liver Cell Biology in Health and Disease. Cells.

[154] Matuz-Mares D., Vázquez-Meza H., Vilchis-Landeros M.M. (2022). NOX as a Therapeutic Target in Liver Disease. Antioxidants.

[155] de Medeiros I.C., de Lima J.G. (2015). Is Nonalcoholic Fatty Liver Disease an Endogenous Alcoholic Fatty Liver Disease?—A Mechanistic Hypothesis. Med. Hypotheses.

[156] Bettaieb A., Jiang J.X., Sasaki Y., Chao T.I., Kiss Z., Chen X., Tian J., Katsuyama M., Yabe-Nishimura C., Xi Y. (2015). Hepatocyte Nicotinamide Adenine Dinucleotide Phosphate Reduced Oxidase 4 Regulates Stress Signaling, Fibrosis, and Insulin Sensitivity during Development of Steatohepatitis in Mice. Gastroenterology.

[157] Zhai L., Pei H., Yang Y., Zhu Y., Ruan S. (2022). NOX4 Promotes Kupffer Cell Inflammatory Response via ROS-NLRP3 to Aggravate Liver Inflammatory Injury in Acute Liver Injury. Aging.

[158] Schwabe R.F., Brenner D.A. (2006). Mechanisms of Liver Injury. I. TNF-Alpha-Induced Liver Injury: Role of IKK, JNK, and ROS Pathways. Am. J. Physiol. Gastrointest. Liver Physiol..

[159] Jeon S.-M. (2016). Regulation and Function of AMPK in Physiology and Diseases. Exp. Mol. Med..

[160] Day E.A., Ford R.J., Steinberg G.R. (2017). AMPK as a Therapeutic Target for Treating Metabolic Diseases. Trends Endocrinol. Metab..

[161] von Loeffelholz C., Coldewey S.M., Birkenfeld A.L. (2021). A Narrative Review on the Role of AMPK on De Novo Lipogenesis in Non-Alcoholic Fatty Liver Disease: Evidence from Human Studies. Cells.

[162] Smith B.K., Marcinko K., Desjardins E.M., Lally J.S., Ford R.J., Steinberg G.R. (2016). Treatment of Nonalcoholic Fatty Liver Disease: Role of AMPK. Am. J. Physiol. Endocrinol. Metab..

[163] Steinberg G.R., Michell B.J., van Denderen B.J.W., Watt M.J., Carey A.L., Fam B.C., Andrikopoulos S., Proietto J., Görgün C.Z., Carling D. (2006). Tumor Necrosis Factor α-Induced Skeletal Muscle Insulin Resistance Involves Suppression of AMP-Kinase Signaling. Cell Metab..

[164] Zhao P., Sun X., Chaggan C., Liao Z., Wong K.I., He F., Singh S., Loomba R., Karin M., Witztum J.L. (2020). An AMPK–Caspase-6 Axis Controls Liver Damage in Nonalcoholic Steatohepatitis. Science.

[165] Henriksen E.J. (2013). Effects of H_2_O_2_ on Insulin Signaling the Glucose Transport System in Mammalian Skeletal Muscle. Methods Enzym..

[166] Blanquicett C., Kang B.-Y., Ritzenthaler J.D., Jones D.P., Hart C.M. (2010). Oxidative Stress Modulates PPARγ in Vascular Endothelial Cells. Free Radic. Biol. Med..

[167] Tendler D., Lin S., Yancy W.S., Mavropoulos J., Sylvestre P., Rockey D.C., Westman E.C. (2007). The Effect of a Low-Carbohydrate, Ketogenic Diet on Nonalcoholic Fatty Liver Disease: A Pilot Study. Dig. Dis. Sci..

[168] Cunha G.M., Guzman G., Correa De Mello L.L., Trein B., Spina L., Bussade I., Marques Prata J., Sajoux I., Countinho W. (2020). Efficacy of a 2-Month Very Low-Calorie Ketogenic Diet (VLCKD) Compared to a Standard Low-Calorie Diet in Reducing Visceral and Liver Fat Accumulation in Patients with Obesity. Front. Endocrinol..

[169] Ministrini S., Calzini L., Nulli Migliola E., Ricci M.A., Roscini A.R., Siepi D., Tozzi G., Daviddi G., Martorelli E.-E., Paganelli M.T. (2019). Lysosomal Acid Lipase as a Molecular Target of the Very Low Carbohydrate Ketogenic Diet in Morbidly Obese Patients: The Potential Effects on Liver Steatosis and Cardiovascular Risk Factors. J. Clin. Med..

[170] Bian H., Hakkarainen A., Lundbom N., Yki-Järvinen H. (2014). Effects of Dietary Interventions on Liver Volume in Humans. Obesity.

[171] Yu H., Jia W., Guo Z. (2014). Reducing Liver Fat by Low Carbohydrate Caloric Restriction Targets Hepatic Glucose Production in Non-Diabetic Obese Adults with Non-Alcoholic Fatty Liver Disease. J. Clin. Med..

[172] Pérez-Guisado J., Muñoz-Serrano A. (2011). The Effect of the Spanish Ketogenic Mediterranean Diet on Nonalcoholic Fatty Liver Disease: A Pilot Study. J. Med. Food.

[173] Paoli A., Tinsley G., Bianco A., Moro T. (2019). The Influence of Meal Frequency and Timing on Health in Humans: The Role of Fasting. Nutrients.

[174] Kirk E., Reeds D.N., Finck B.N., Mayurranjan M.S., Patterson B.W., Klein S. (2009). Dietary Fat and Carbohydrates Differentially Alter Insulin Sensitivity During Caloric Restriction. Gastroenterology.

[175] Browning J.D., Baker J.A., Rogers T., Davis J., Satapati S., Burgess S.C. (2011). Short-Term Weight Loss and Hepatic Triglyceride Reduction: Evidence of a Metabolic Advantage with Dietary Carbohydrate Restriction. Am. J. Clin. Nutr..

[176] Ruderman N.B., Julia Xu X., Nelson L., Cacicedo J.M., Saha A.K., Lan F., Ido Y. (2010). AMPK and SIRT1: A Long-Standing Partnership?. Am. J. Physiol. Endocrinol. Metab..

[177] Draznin B., Wang C., Adochio R., Leitner J., Cornier M.-A. (2012). Effect of Dietary Macronutrient Composition on AMPK and SIRT1 Expression and Activity in Human Skeletal Muscle. Horm. Metab. Res..

[178] Jani S., da Eira D., Stefanovic M., Ceddia R.B. (2022). The Ketogenic Diet Prevents Steatosis and Insulin Resistance by Reducing Lipogenesis, Diacylglycerol Accumulation and Protein Kinase C Activity in Male Rat Liver. J. Physiol..

[179] Mardinoglu A., Wu H., Bjornson E., Zhang C., Hakkarainen A., Räsänen S.M., Lee S., Mancina R.M., Bergentall M., Pietiläinen K.H. (2018). An Integrated Understanding of the Rapid Metabolic Benefits of a Carbohydrate-Restricted Diet on Hepatic Steatosis in Humans. Cell Metab..

[180] Li H., Zhang J., Jia W. (2013). Fibroblast Growth Factor 21: A Novel Metabolic Regulator from Pharmacology to Physiology. Front. Med..

[181] Li H., Fang Q., Gao F., Fan J., Zhou J., Wang X., Zhang H., Pan X., Bao Y., Xiang K. (2010). Fibroblast Growth Factor 21 Levels Are Increased in Nonalcoholic Fatty Liver Disease Patients and Are Correlated with Hepatic Triglyceride. J. Hepatol..

[182] Dushay J., Chui P.C., Gopalakrishnan G.S., Varela–Rey M., Crawley M., Fisher F.M., Badman M.K., Martinez–Chantar M.L., Maratos–Flier E. (2010). Increased Fibroblast Growth Factor 21 in Obesity and Nonalcoholic Fatty Liver Disease. Gastroenterology.

[183] Bueno N.B., de Melo I.S.V., de Oliveira S.L., da Rocha Ataide T. (2013). Very-Low-Carbohydrate Ketogenic Diet *v.* Low-Fat Diet for Long-Term Weight Loss: A Meta-Analysis of Randomised Controlled Trials. Br. J. Nutr..

